# A longitudinal assessment of antimicrobial susceptibility among important pathogens collected as part of the Tigecycline Evaluation and Surveillance Trial (T.E.S.T.) in France between 2004 and 2012

**DOI:** 10.1186/2047-2994-3-36

**Published:** 2014-12-01

**Authors:** Vincent Cattoir, Michael J Dowzicky

**Affiliations:** CHU de Caen, Microbiologie & Centre National de Référence de la Résistance aux Antibiotiques (laboratoire associé Entérocoques et résistances particulières des bactéries à Gram positif), Caen, France; Pfizer Inc, Collegeville, PA USA; CHU de Caen, Service de Microbiologie - Niveau 3, Avenue de la Côte de Nacre-CS30001, 14033 Caen, Cedex 9, France

**Keywords:** France, Antimicrobial resistance, Antimicrobial surveillance, Multidrug resistance, MDR, Tigecycline

## Abstract

**Background:**

Clinically important Gram-positive and -negative isolates were collected from patients in France between 2004 and 2012 as a part of the Tigecycline Evaluation and Surveillance Trial.

**Methods:**

MICs were determined using methodology described by the Clinical and Laboratory Standards Institute.

**Results:**

In total, 17,135 isolates were contributed by 29 medical centres; respiratory (25.1%) and cardiovascular (20.3%) sources predominated. High susceptibility was observed among *Enterococcus* spp. and *Staphylococcus aureus* (including methicillin-resistant *S. aureus* [MRSA]) to linezolid (100%), tigecycline (≥99.8%) and vancomycin (≥94.6%). The percentage of MRSA decreased from 34.3% in 2004 to 20.0% in 2009 before increasing to 34.7% in 2012. Vancomycin, linezolid, levofloxacin and carbapenems were highly active (≥99.6%) against *Streptococcus pneumoniae*; 3.2% were PRSP. *Escherichia coli* showed peak susceptibility to the carbapenems (≥99.9%), tigecycline (99.3%) and amikacin (97.9%); significant (p < 0.01) decreases in susceptibility were observed for ampicillin, cefepime and ceftriaxone between 2004 and 2012. ESBL production among *E. coli* increased from 3.0% (2004) to 14.9% (2012). High susceptibility was noted among *Haemophilus influenzae* to levofloxacin (100%), amoxicillin-clavulanate (99.2%), carbapenems (≥98.7%) and ceftriaxone (98.5%); β-lactamase production fluctuated with no notable trend between 18.1% (2007) and 27.7% (2011). *Klebsiella* spp. were highly susceptible to carbapenems (≥99.6%) and amikacin (≥96.4%); significant (p < 0.01) decreases in amoxicillin-clavulanate, cefepime, ceftriaxone, levofloxacin, piperacillin-tazobactam and tigecycline susceptibility were observed among *K. pneumoniae* between 2004 and 2012. Only imipenem was highly active (96.5% susceptible) against *Acinetobacter baumannii*. Imipenem and amikacin (87.7% and 87.1% susceptible) were the most active agents against *P. aeruginosa*; 10.2% of isolates were categorized as multidrug resistant.

**Conclusions:**

Carbapenems, linezolid, tigecycline and vancomycin conserved good in vitro activity against most pathogens (according to their spectrum of activity) in France between 2004 and 2012.

**Electronic supplementary material:**

The online version of this article (doi:10.1186/2047-2994-3-36) contains supplementary material, which is available to authorized users.

## Background

France is home to one of the highest rates of antibiotic consumption and antimicrobial resistance in Europe [1], and has experienced rapidly changing trends of antimicrobial resistance in recent years. The European Antimicrobial Resistance Surveillance Network (EARS-Net) has reported significantly increasing levels of resistance in France [2], where 10.8% of *Escherichia coli* and 23.7% of *Klebsiella pneumoniae* isolates were reported to be intermediate or resistant to third-generation cephalosporins in 2012 (as compared with 1.9% in 2002 and 5.1% in 2005, respectively) [3]. Several programmes have been initiated to combat these increasing levels of resistance, including measures to control transmission of resistant pathogens, to promote the use of alcohol-based hand-rub solution in hospitals, to control/prevent the spread of emerging multidrug-resistant (MDR) organisms (i.e., vancomycin-resistant enterococci [VRE], carbapenemase-producing Enterobacteriaceae) and to decrease antibiotic consumption [4]. These efforts have paid at least some dividends: declining levels of antimicrobial resistance have been reported in recent years among French isolates of *Streptococcus pneumoniae* to penicillin (from 36.2% in 2005 to 23.4% in 2012) and *Staphylococcus aureus* to methicillin (from 33.4% in 2001 to 19.1% in 2012) [3].

Tigecycline is a broad-spectrum antimicrobial agent which has been indicated for use in the treatment of complicated skin and skin structure infections (cSSTIs) and complicated intra-abdominal infections (cIAIS) (and in the USA, community-acquired bacterial pneumonia) [5]. The Tigecycline Evaluation and Surveillance Trial (T.E.S.T.) is a global surveillance study which commenced in 2004, with the intention of monitoring the activity of the broad-spectrum glycylcycline tigecycline and a panel of comparator agents against an array of clinically important Gram-positive and Gram-negative organisms. In this study, we examine the activity of tigecycline and comparators against clinically important Gram-positive and Gram-negative pathogens collected from community and nosocomial patients in France between 2004 and 2012. This manuscript serves as an update to Rodloff et al. [6], who described a collection of isolates from France, Germany, Italy, Spain and the U.K. collected as a part of T.E.S.T. between 2004 and 2006, as well as Nørskov-Lauritsen et al. [7], who presented data on European isolates (including France) collected between 2004 and 2007.

## Methods

Between 2004 and 2012 there were 29 centres in France. The majority of these centres were university hospitals. No centres contributed in all 9 study years. Three centres contributed in 8 years, two in 7 years, four in 6 years, six in 5 years, three in 4 years, three in 3 years, five in 2 years, and three in a single year.

### Bacterial isolates

Each centre was required to submit a minimum of 65 Gram-positive isolates and 135 Gram-negative isolates, including at least 25 *S. aureus*, 15 *Enterococcus* spp., 15 *S. pneumoniae*, 10 *Streptococcus agalactiae*, 25 *Klebsiella* spp., 25 *E. coli*, 25 *Enterobacter* spp., 20 *Pseudomonas aeruginosa*, 15 *Acinetobacter* spp., 15 *H. influenzae* and 10 *Serratia* spp. isolates. Each submitted isolate had to be considered by the contributing centre to be of clinical significance as the probable causative agent of a hospital- or community-acquired infection. All body sites were considered acceptable isolate sources for this study, including body fluid, central nervous system, cardiovascular system, gastro-intestinal, genito-urinary (no more than 25% of isolates from any centre), head, ears, eyes, nose and throat, integument, lymph, muscular, reproductive, respiratory, skeletal or medical instruments (i.e. catheters, drains, forceps, probes). No banked or stored isolates or duplicate isolates from a single patient were accepted into the T.E.S.T. study. Isolate inclusion was independent of patient age, sex, antimicrobial use and/or medical history.

All isolates were sent to a single reference laboratory, International Health Management Associates (IHMA, Schaumburg, IL), which was responsible for organism collection and transport and organism identification confirmation and development. IHMA also undertook creation and management of a centralized isolate database. Quality control (QC) checks were carried out by IHMA on approximately 10% of isolates annually.

### Antimicrobial susceptibility testing

Minimum inhibitory concentrations (MICs) were determined locally using broth microdilution methodology as described by the Clinical and Laboratory Standards Institute (CLSI) [8] using either MicroScan® panels (Dade Behring Inc., CA, USA) or Sensititre® plates (TREK Diagnostic Systems, West Sussex, England). The test panel for the T.E.S.T. study included amikacin (AMK), amoxicillin-clavulanate (AMC), ampicillin (AMP), cefepime (CFP), ceftazidime (CTZ), ceftriaxone (CRO), imipenem (IMP), levofloxacin (LEV), linezolid (LZD), meropenem (MER), minocycline (MIN), penicillin (PEN), piperacillin-tazobactam (PTZ), tigecycline (TIG) and vancomycin (VAN). Imipenem was replaced in 2006 by meropenem due to stability issues associated with imipenem and MicroScan® panels were replaced by Sensititre® the same year. After 2006, the test panel for *S. pneumoniae* also included azithromycin (AZI), clarithromycin (CLA), erythromycin (ERY) and clindamycin (CLI). Clinical categorization was done using the 2013 breakpoints established by the European Committee on Antimicrobial Susceptibility Testing (EUCAST) [9]. Data are included in the tables only when interpretive breakpoints are available.

Extended-spectrum β-lactamase (ESBL) production among *E. coli* and *Klebsiella* spp. was identified by IHMA using cefotaxime (30 μg), cefotaxime-clavulanic acid (30/10 μg), ceftazidime (30 μg), and ceftazidime-clavulanic acid (30/10 μg) discs [10]. A positive ESBL result was designated by an increase of ≥5 mm in the inhibition zone on the combination disc compared with the corresponding cephalosporin disc. Discs were manufactured by Oxoid, Inc. (Ogdensburg, NY, USA); Mueller-Hinton agar was produced by Remel, Inc. (Lenexa, KS, USA). *H. influenzae* isolates were tested for β-lactamase production using locally preferred methodologies. Multidrug resistance was defined as resistance to three or more classes of antimicrobial agent, and only included antimicrobials with available breakpoints. For *A. baumannii* antimicrobials classes (and agents) included in the analysis were aminoglycosides [AMK], carbapenems [IMP or MER], and fluoroquinolones [LEV]. For *P. aeruginosa* antimicrobial classes (and agents) included in the analysis were aminoglycosides [AMK], β-lactams [CFP, CTZ, PTZ], carbapenems [IMP or MER], and fluoroquinolones [LEV].

Daily QC testing was performed using QC strains *Enterococcus faecalis* ATCC 29212, *S. aureus* ATCC 29213, *S. pneumoniae* ATCC 49619, *E. coli* ATCC 25922, *P. aeruginosa* ATCC 27853 and *H. influenzae* ATCC 49247 and ATCC 49766, as appropriate. QC strains used for ESBL testing were *K. pneumoniae* ATCC 700603 (ESBL-positive) and *E. coli* ATCC 25922 (ESBL-negative), while *P. aeruginosa* (ATCC 27853) was used for the QC of ceftazidime and cefotaxime discs. Information on T.E.S.T. study protocols can be found online [5].

Longitudinal data were examined for statistically significant changes in susceptibility between 2004 and 2012 using the Cochran Armitage Trend Test. A positive change reflected a statistically significant decrease in susceptibility, while a negative change indicated that susceptibility had increased significantly. A p < 0.01 was used in this analysis as a cut-off value for statistical significance (a significance value of p < 0.05 was not used here as computing a high volume of statistical tests can lead to significant results purely by chance; setting a lower significance value greatly reduces the chance of this happening).

## Results

Isolates were collected from 29 centres in France between 2004 and 2012 (eight in 2004, six in 2005, 12 in 2006, 16 in 2007, 21 in 2008, 20 in 2009, 15 in 2010, five in 2011 and 23 in 2012) as a part of the T.E.S.T surveillance study.

### Gram-positive pathogens

#### Enterococcus spp

Between 2004 and 2012, 969 isolates of *E. faecalis* and 332 *Enterococcus faecium* isolates were examined as a part of the T.E.S.T. study (Table 1). Both species were highly susceptible to linezolid (both 100%), tigecycline (99.8% and 100%, respectively) and vancomycin (99.3% and 94.6%, respectively). *E. faecalis* were also highly susceptible to amoxicillin-clavulanate, ampicillin and imipenem (>96%), while *E. faecium* were not (≤25% susceptible). Decreases in *E. faecalis* susceptibility between 2004 and 2012 to amoxicillin-clavulanate (100% to 96.7%) and ampicillin (100% to 95.4%) were small but statistically significant (p < 0.01 and p < 0.001, respectively). Of note, vancomycin resistance was observed in 0.7% of *E. faecalis* isolates (increasing from 0.0% in 2004 to 1.3% in 2012) and 5.4% of *E. faecium* isolates (increasing from 0.0% in 2004 to 4.3% in 2012) between 2004 and 2012 in France. Linezolid and tigecycline activity were unaffected by vancomycin resistance (Table 2).

**Table 1:** **Minimum inhibitory concentrations (MIC**_**50**_ **, MIC**_**90**_ **, MIC range [mg/L]) and antimicrobial susceptibility (%S) of clinically important Gram-positive and Gram-negative isolates**

Pathogen	N	MIC _50_	MIC _90_	MIC Range	%S
**Gram-positive**					
***E. faecalis***					
AMC	969	0.5	1	≤0.03 - ≥16	99.1
AMP	969	1	2	≤0.06 - ≥32	98.8
IMP	137	1	4	≤0.12 - 16	96.4
LZD	969	2	2	≤0.5 - 4	100
TIG	969	0.12	0.25	≤0.008 - 0.5	99.8
VAN	969	1	2	0.25 - ≥64	99.3
***E. faecium***					
AMC	332	≥16	≥16	0.06 - ≥16	25.0
AMP	332	≥32	≥32	≤0.06 - ≥32	22.3
IMP	29	≥32	≥32	2 - ≥32	20.7
LZD	332	2	2	≤0.5 - 2	100
TIG	332	0.06	0.25	0.03 - 0.25	100
VAN	332	1	2	0.25 - ≥64	94.6
***S. aureus***					
LEV	2229	0.25	16	≤0.06 - ≥64	71.7
LZD	2229	2	2	≤0.5 - 4	100
MIN	2229	≤0.25	0.5	≤0.25 - ≥16	93.7
PEN	2229	8	≥16	≤0.06 - ≥16	14.4
TIG	2229	0.12	0.25	≤0.008 - 0.5	100
VAN	2229	1	1	≤0.12 - 2	100
***S. agalactiae***					
LEV	859	0.5	1	≤0.06 - 32	97.1
LZD	859	1	1	≤0.5 - 2	100
MIN	859	8	≥16	≤0.25 - ≥16	15.4
PEN	859	≤0.06	0.12	≤0.06 - 0.12	100
TIG	859	0.06	0.12	0.015 - 0.25	100
VAN	859	0.5	0.5	≤0.12 - 1	100
***S. pneumoniae***					
AMP	990	≤0.06	2	≤0.06 - ≥32	68.2
AZI	872	0.12	≥128	≤0.03 - ≥128	56.5
CRO	990	0.06	1	≤0.03 - 16	79.1
CLA	872	0.06	≥128	≤0.015 - ≥128	57.0
CLI	872	0.06	≥128	≤0.015 - ≥128	65.0
ERY	872	0.12	≥128	≤0.015 - ≥128	56.4
IMP	120	≤0.12	0.25	≤0.12 - 0.5	100
LEV	990	1	1	≤0.06 - ≥64	99.6
LZD	990	1	1	≤0.5 - 4	99.9
MER	870	≤0.12	0.5	≤0.12 - ≥32	99.9
MIN	990	1	8	≤0.25 - ≥16	47.6
PEN	990	≤0.06	2	≤0.06 - ≥16	51.3
VAN	990	0.25	0.5	≤0.12 - 1	100
**Gram-negative**					
***E. aerogenes***					
AMK	561	2	8	≤0.5 - 64	95.9
CFP	561	≤0.5	2	≤0.5 - ≥64	87.0
CRO	561	0.5	32	≤0.06 - ≥128	56.7
IMP	81	0.5	1	≤0.06 - 4	97.5
LEV	561	0.06	≥16	≤0.008 - ≥16	76.5
MER	480	≤0.06	0.12	≤0.06 - 8	98.3
PTZ	561	8	64	0.25 - ≥256	59.5
TIG	561	0.5	2	0.12 - 16	87.0
***E. cloacae***					
AMK	1665	1	4	≤0.5 - ≥128	96.7
CFP	1665	≤0.5	8	≤0.5 - ≥64	66.5
CRO	1665	1	≥128	≤0.06 - ≥128	50.7
IMP	226	0.5	1	≤0.06 - 8	99.1
LEV	1665	0.06	≥16	≤0.008 - ≥16	73.1
MER	1439	≤0.06	0.25	≤0.06 - ≥32	99.4
PTZ	1665	4	≥256	≤0.06 - ≥256	59.3
TGC	1665	0.5	2	0.06 - 16	85.0
***E. coli***					
AMK	2284	2	4	≤0.5 - ≥128	97.9
AMC	2284	8	32	0.25 - ≥64	70.8
AMP	2284	≥64	≥64	≤0.5 - ≥64	38.4
CFP	2284	≤0.5	8	≤0.5 - ≥64	84.3
CRO	2284	≤0.06	64	≤0.06 - ≥128	84.0
IMP	324	0.25	0.5	≤0.06 - 2	100
LEV	2284	0.03	≥16	≤0.008 - ≥16	79.9
MER	1960	≤0.06	≤0.06	≤0.06 - 4	99.9
PTZ	2284	2	16	≤0.06 - ≥256	89.0
TGC	2284	0.25	0.5	≤0.008 - 2	99.3
***H. influenzae***					
AMC	1191	0.5	1	≤0.12 - 16	99.2
AMP	1191	≤0.5	32	≤0.5 - ≥64	75.6
CRO	1191	≤0.06	≤0.06	≤0.06 - 4	98.5
IMP	156	0.25	0.5	≤0.06 - 4	98.7
LEV	1191	0.015	0.015	≤0.008 - 1	100
MER	1035	≤0.06	0.12	≤0.06 - 0.5	100
MIN	1191	≤0.5	1	≤0.5 - 16	90.8
***K. oxytoca***					
AMK	695	1	4	≤0.5 - ≥128	98.7
AMC	695	2	32	0.25 - ≥64	79.7
CFP	695	≤0.5	2	≤0.5 - ≥64	89.2
CRO	695	≤0.06	8	≤0.06 - ≥128	83.3
IMP	102	0.25	0.5	≤0.06 - 1	100
LEV	695	0.06	1	≤0.008 - ≥16	90.5
MER	593	≤0.06	≤0.06	≤0.06 - ≥32	99.7
PTZ	695	2	≥256	≤0.06 - ≥256	83.3
TGC	695	0.25	1	0.015 - 8	95.4
***K. pneumoniae***					
AMK	1524	1	4	≤0.5 - ≥128	96.4
AMC	1524	4	32	0.5 - ≥64	72.6
CFP	1524	≤0.5	32	≤0.5 - ≥64	79.4
CRO	1524	≤0.06	≥128	≤0.06 - ≥128	77.2
IMP	211	0.25	0.5	≤0.06 - 2	100
LEV	1524	0.06	8	≤0.008 - ≥16	82.2
MER	1313	≤0.06	≤0.06	≤0.06 - ≥32	99.6
PTZ	1524	2	64	0.12 - ≥256	81.3
TGC	1524	0.5	2	0.06 - 16	87.6
***S. marcescens***					
AMK	895	2	4	≤0.5 - ≥128	97.1
CFP	895	≤0.5	1	≤0.5 - ≥64	94.4
CRO	895	0.25	8	≤0.06 - ≥128	80.4
IMP	118	0.5	1	≤0.06 - 4	96.6
LEV	895	0.12	2	0.015 - ≥16	87.2
MER	777	≤0.06	0.12	≤0.06 - ≥32	98.7
PTZ	895	2	16	≤0.06 - ≥256	88.7
TGC	895	1	2	0.015 - 8	80.1
***A. baumannii***					
AMK	1161	4	64	≤0.5 - ≥128	75.6
IMP	170	0.5	2	≤0.06 - ≥32	96.5
LEV	1161	0.25	8	≤0.008 - ≥16	56.8
MER	991	0.5	8	≤0.06 - ≥32	83.7
***P. aeruginosa***					
AMK	1780	4	16	≤0.5 - ≥128	87.1
CFP	1780	4	32	≤0.5 - ≥64	77.5
CTZ	1780	≤8	32	≤8 - ≥64	75.6
IMP	260	1	8	0.12 - ≥32	87.7
LEV	1780	1	≥16	≤0.008 - ≥16	58.3
MER	1520	0.5	8	≤0.06 - ≥32	75.4
PTZ	1780	8	≥256	0.12 - ≥256	72.5

AMK, amikacin; AMC, amoxicillin-clavulanate; AMP, ampicillin; CFP, cefepime; CTZ, ceftazidime; CRO, ceftriaxone; IMP, imipenem; LEV, levofloxacin; LZD, linezolid; MER, meropenem; MIN, minocycline; PEN, penicillin; PTZ, piperacillin-tazobactam; TIG, tigecycline; VAN, vancomycin.

**Table 2:** **MIC**_**90**_ **(mg/L), antimicrobial susceptibility (%S) and statistically significant changes in susceptibility among resistant pathogen phenotypes**

Pathogen	Antimicrobial	MIC _90_	%S	Significance ^a^
**Gram-positive**				
*E. faecium*, VRE (n = 18 [0/18])	AMC	≥16	16.7	N.S.
	AMP	≥32	16.7	N.S.
	LZD	2	100	-
	TIG	0.25	100	-
	VAN	≥64	0.0	-
*S. aureus*, MRSA (n = 631 [77/554])	LEV	32	13.2	N.S.
	LZD	2	100	-
	MIN	0.5	93.5	p < 0.001 (−)
	PEN	≥16	0.0	-
	TIG	0.25	100	-
	VAN	1	100	-
*S. pneumoniae*, PRSP (n = 32; 31^b^)	AMP	8	0.0	-
	AZI	≥128	19.4	N.S.
	CRO	2	6.3	N.S.
	CLA	≥128	19.4	N.S.
	CLI	≥128	32.3	p < 0.01 (−)
	ERY	≥128	19.4	N.S.
	LEV	2	96.9	N.S.
	LZD	1	100	-
	MER	1	96.9	N.S.
	MIN	≥16	18.8	N.S.
	PEN	4	0.0	-
	VAN	0.5	100	-
**Gram-negative**				
*E. coli*, ESBL (n = 275 [17/258])	AMK	8	90.5	p < 0.001 (−)
	AMC	32	36.7	p < 0.001 (−)
	AMP	≥64	0.0	-
	CFP	≥64	4.7	N.S.
	CRO	≥128	0.0	-
	IMP	0.5	100	-
	LEV	≥16	37.8	p < 0.01 (−)
	MER	≤0.06	100	-
	PTZ	64	72.4	p < 0.01 (−)
	TIG	0.5	98.9	N.S.
*H. influenzae*, BL-Pos (n = 269 [32/237])	AMC	2	98.1	N.S.
	AMP	≥64	0.4	N.S.
	CRO	≤0.06	97.4	N.S.
	IMP	1	100	-
	LEV	0.015	100	-
	MER	0.12	100	-
	MIN	1	92.6	N.S.
*K. pneumoniae*, ESBL (n = 274 [19/255])	AMK	16	85.0	N.S.
	AMC	32	16.1	N.S.
	CFP	≥64	7.3	N.S.
	CRO	≥128	1.8	N.S.
	IMP	0.5	100	N.S.
	LEV	≥16	29.6	N.S.
	MER	0.12	98.4	-
	PTZ	≥256	39.4	N.S.
	TIG	2	78.1	N.S.

^a^A negative (−) change in significance indicates an increase in susceptibility; N.S., not significant. A cut-off of p < 0.1 was used for statistical significance testing.

Values given in square parentheses refer to the number of isolates tested against imipenem and meropenem, respectively (and, where different, ampicillin [^b^]).

Only seven vancomycin-resistant *E. faecalis* were collected during this study; data not presented.

#### S. aureus

All (N = 2229) *S. aureus* isolates were susceptible to linezolid, tigecycline and vancomycin, including MRSA isolates, while 93.7% were susceptible to minocycline (Table 1). The percentage of *S. aureus* identified as MRSA in France decreased from 34.3% in 2004 to 20.0% in 2009, but increased to 34.7% in 2012; the average MRSA rate over the total course of the study was 28.3% (Table 3). There was a statistically significant (p < 0.001) increase in minocycline susceptibility among MRSA over the study duration (Table 2). Methicillin resistance had no impact on the activity of linezolid, minocycline, tigecycline or vancomycin.

**Table 3:** **Percentages of resistant phenotypes among Gram-positive and Gram—negative isolates by year, 2004–2012**

Pathogen		2004	2005	2006	2007	2008	2009	2010	2011	2012	2004-12
	N	n (%)	n (%)	n (%)	n (%)	n (%)	n (%)	n (%)	n (%)	n (%)	n (%)
**Gram-positive**											
*E. faecalis*, VRE	969	0 (0.0)	0 (0.0)	1 (1.5)	0 (0.0)	0 (0.0)	2 (1.1)	1 (0.7)	1 (3.8)	2 (1.3)	7 (0.7)
*E. faecium*, VRE	332	0 (0.0)	0 (0.0)	0 (0.0)	2 (4.9)	8 (10.8)	2 (3.3)	3 (5.5)	1 (6.7)	2 (4.3)	18 (5.4)
*S. aureus*, MRSA	2229	34 (34.3)	20 (31.7)	40 (30.5)	88 (29.6)	135 (28.2)	77 (20.0)	75 (26.1)	15 (23.4)	147 (34.7)	631 (28.3)
*S. pneumoniae*, PRSP	990	0 (0.0)	1 (3.4)	3 (4.6)	3 (2.0)	9 (4.5)	7 (4.0)	6 (4.1)	0 (0.0)	3 (2.0)	32 (3.2)
**Gram-negative**											
*A. baumannii*, MDR	1161	0 (0.0)	0 (0.0)	2 (2.0)	7 (4.9)	12 (5.2)	12 (5.2)	13 (6.7)	1 (3.0)	7 (4.7)	54 (4.7)
*E. coli*, ESBL	2284	3 (3.0)	2 (3.7)	8 (4.6)	18 (6.5)	58 (11.8)	75 (17.5)	46 (13.9)	13 (16.9)	52 (14.9)	275 (12.0)
*H. influenzae*, BL-Pos	1191	13 (23.2)	6 (23.1)	17 (27.4)	30 (18.1)	53 (21.1)	56 (25.0)	36 (23.7)	13 (27.7)	45 (21.7)	269 (22.6)
*K. pneumoniae*, ESBL	1524	5 (7.6)	5 (9.3)	9 (10.3)	20 (12.3)	64 (18.6)	47 (16.4)	58 (24.9)	11 (22.0)	55 (23.0)	274 (18.0)
*P. aeruginosa*, MDR	1780	2 (2.5)	0 (0.0)	17 (11.2)	23 (10.7)	39 (10.2)	37 (11.0)	33 (12.5)	9 (14.8)	21 (8.1)	181 (10.2)

ESBL, extended-spectrum β-lactamase; BL-Pos, β-lactamase-positive; MDR, multidrug-resistant; MRSA, methicillin-resistant *S. aureus*; PRSP, penicillin-resistant *S. pneumoniae*; VRE, vancomycin-resistant *Enterococcus.*

Results do not exactly match those presented by Nørskov-Lauritsen et al. [7] due to subsequent addition and deletion of isolates from the T.E.S.T. database.

#### S. agalactiae

*S. agalactiae* (N = 859) were highly susceptible to most agents on the TEST panel where breakpoints exist, the notable exception being minocycline (against which only 15.4% of isolates were susceptible) (Table 1).

#### S. pneumoniae

*S. pneumoniae* (N = 990) were highly susceptible to vancomycin (100%), linezolid (99.9%) and levofloxacin (99.6%). Imipenem and meropenem were also highly active (100% and 99.9% susceptibility, respectively), although only tested against a subset of isolates (n = 120 and n = 870) (Table 1). A MIC_90_ of 0.06 mg/L was reported for tigecycline (no tigecycline breakpoints are available). Statistically significant changes in susceptibility were observed between 2004 and 2012 for clindamycin (increasing from 52.3% to 67.4%; p < 0.01) and minocycline (decreasing from 55.8 to 50.3%; p < 0.01) (Additional file 1: Table S1). No penicillin-resistant *S. pneumoniae* (PRSP) were collected in 2004 or 2011 (Table 3). The highest rate of penicillin resistance was reported in 2006 (4.6%). The PRSP rate over the 2004–2012 period in France was 3.2% (Table 3). A statistically significant (p < 0.01) increase in clindamycin susceptibility was observed among PRSP isolates (Table 2). Susceptibly to levofloxacin, linezolid, meropenem, and vancomycin were largely unaffected by penicillin resistance (Table 2). The MIC_90_ for tigecycline was 0.03 mg/L against penicillin-resistant isolates.

### Gram-negative pathogens

#### Enterobacter spp

Meropenem, imipenem and amikacin were the most active agents against *Enterobacter* spp., with 98.3% (n = 480), 97.5% (n = 81) and 95.9% of *E. aerogenes* (N = 561) and 99.4% (n = 1439), 99.1% (n = 226) and 96.7% of *E. cloacae* (N = 1665) isolates susceptible, respectively (Table 1). *E. aerogenes* and *E. cloacae* were 87.0% and 85.0% susceptible to tigecycline, respectively. No statistically significant changes in susceptibility over time were reported for *Enterobacter* spp..

#### E. coli

*E. coli* (N = 2284) were highly susceptible to imipenem (100%; n = 324), meropenem (99.9%; n = 1960), tigecycline (99.3%) and amikacin (97.9%). Statistically significant decreases in susceptibility were observed to ampicillin (p < 0.001; 55.4% to 33.2%), cefepime (p < 0.0001; 97.0% to 81.7%) and ceftriaxone (p < 0.0001; 96.0% to 81.1%) between 2004 and 2012 (Additional file 1: Table S1). The percentage of ESBL-positive *E. coli* isolates increased from 3.0% in 2004 to 14.9% in 2012, reaching a maximum of 17.5% in 2009 (Table 3). Statistically significant increases in susceptibility were observed among ESBL-positive *E. coli* to amikacin (p < 0.001), amoxicillin-clavulanate (p < 0.001), levofloxacin (p < 0.01) and piperacillin-tazobactam (p < 0.01) (Table 2). Carbapenem and tigecycline activity were not impacted by ESBL production (Table 2).

#### H. influenzae

All isolates of *H. influenzae* (N = 1191) were susceptible to levofloxacin and meropenem (n = 1035); susceptibility was also high to amoxicillin-clavulanate (99.2%), imipenem (98.7%; n = 156) and ceftriaxone (98.5%). The MIC_90_ of tigecycline was 0.25 mg/L. The percentage of β-lactamase positive isolates did not change notably between 2004 and 2012 (Table 3). As expected, the in vitro activity of ampicillin was dramatically reduced against β-lactamase-positive *H. influenzae* (Table 2).

#### Klebsiella spp

Both *K. oxytoca* (N = 695) and *K. pneumoniae* (N = 1524) were fully susceptible to imipenem (n = 102 and 211, respectively). High levels of susceptibility were also reported for meropenem (99.7% [n = 593] and 99.6% [n = 1313], respectively) and amikacin (98.7% and 96.4%, respectively) (Table 1). Statistically significant decreases in susceptibility were observed among *K. pneumoniae* to amoxicillin-clavulanate (p < 0.0001; 84.8% to 69.5%), cefepime (p < 0.0001; 95.5% to 69.9%), ceftriaxone (p < 0.0001; 90.9% to 69.9%), levofloxacin (p < 0.0001; 93.9% to 77.4%), piperacillin-tazobactam (p < 0.0001; 95.5% to 82.4%) and tigecycline (p < 0.01; 93.9% to 84.9%) over the 2004–2012 interval (Additional file 1: Table S1). ESBL production among *K. pneumoniae* isolates increased from 7.6% in 2004 to 23.0% in 2012 (Table 3). Carbapenem activity was not impacted by ESBL production, while amikacin and tigecycline activity decreased by approximately 10% (Table 2). No statistically significant changes in susceptibility were reported for *K. oxytoca*.

#### S. marcescens

The most active antimicrobial agents in this study against *S. marcescens* (N = 895) were meropenem (98.7% susceptible; n = 777), amikacin (97.1% susceptible), imipenem (96.6% susceptible; n = 118) and cefepime (94.4% susceptible). No statistically significant changes in susceptibility over time were reported.

#### A. baumannii

The most active agent against *A. baumannii* (N = 1161) was imipenem (96.5% susceptible; n = 170), although data are only available up to 2007 (Table 1). No breakpoint is available for tigecycline, for which a MIC_90_ of 1 mg/L was recorded. Multidrug resistance was reported among 4.7% of *A. baumannii* isolates between 2004 and 2012, reaching a maximum of 6.7% in 2010 (Table 3).

#### P. aeruginosa

Imipenem (n = 260) and amikacin were the most active agents against *P. aeruginosa* with 87.7% and 87.1% susceptibility, respectively (Table 1). A total of 10.2% of *P. aeruginosa* isolates were MDR, ranging from 0.0% in 2005 to 14.8% in 2011 (Table 3).

## Discussion

This report updates data previously presented by Rodloff et al. [6] for France (as well as Germany, Italy, Spain and the U.K.) between 2004 and 2006 and Nørskov-Lauritsen et al. [7] for data collected between 2004 and 2007. The data described in their reports are included in the dataset described in this manuscript. Susceptibility results are difficult to compare between these two earlier reports and the current study as CLSI interpretive breakpoints were used in Rodloff et al. [6] and Nørskov-Lauritsen et al. [7] while EUCAST breakpoints have been used in the current manuscript. No vancomycin-resistant enterococci were reported in either earlier study in France; however, small percentages of vancomycin-resistant *E. faecalis* (0.7%) and *E. faecium* (5.4%) were collected in the current study. As the data show, the majority of vancomycin-resistant enterococci were collected during or after 2008 (three isolates were collected in 2006 and 2007 but were not reported by Rodloff et al. [6] and Nørskov-Lauritsen et al. [7] as they were entered into the database after the data cut-offs for these publications). Rates of MRSA were comparable between the three reports (28.3% in the current study, 28.3% in Rodloff et al. [6], and 31.5% in Nørskov-Lauritsen et al. [7]); however, the rate of penicillin-resistant *S. pneumoniae* was lower in the current study when compared with Nørskov-Lauritsen et al. [7] (3.2% and 16.8%, respectively). No *S. pneumoniae* data was presented by Rodloff et al. [6]. This difference is likely due in part to the use of CLSI breakpoints by Nørskov-Lauritsen et al. (resistance breakpoint ≥2 mg/L, compared to ≥4 mg/L used by EUCAST); the removal of 236 *S. pneumoniae* isolates from the T.E.S.T. database whose MICs could not be verified (i.e., isolates which could not be revived for retesting or which died on transport from the contributing centre to IHMA) may have also influenced this PRSP difference.

ESBL production among *E. coli* and *K. pneumoniae* was higher in the current study; 12.0% and 18.0% compared with 4.9% and 9.5% and 5.1% and 9.8% in Rodloff et al. [6] and Nørskov-Lauritsen et al. [7], respectively. As rates of ESBLs were higher in the later years of this study (2008 onwards) this difference is not unexpected. Rates of multidrug-resistant *A. baumannii* and β-lactamase producing *H. influenzae* were similar between the current report and Nørskov-Lauritsen et al. [7]. (approximately 5% and 22%, respectively), although the definition of MDR *A. baumannii* in Nørskov-Lauritsen et al. [7]. also included cephalosporins. Data on multidrug-resistant *A. baumannii* and *H. influenzae* were not reported by Rodloff et al. [6]. As the isolates presented by Rodloff et al. [6] and Nørskov-Lauritsen et al. [7] are also included in this report comparisons between these three reported must be treated with some caution. However, the increases in rates of vancomycin-resistant enterococci, and ESBL-producing *E. coli* and *K. pneumoniae* are cause for concern and warrant further monitoring.

One factor that could influence the difference in resistance rates between the reports is the presence of centre specific outbreaks. Outbreaks of resistant pathogens have been described in several medical centres in France in recent years, caused by carbapenemase-producing [11] or metallo-β-lactamase-producing *K. pneumoniae* [12], MDR *A. baumannii* [13], glycopeptide-intermediate *S. aureus* [14] and vancomycin-resistant enterococci [4, 15]. These outbreaks were controlled with infection control measures, including strict enforcement of hygiene precautions, limiting transfer of patients to other wards, isolating infected patients with dedicated staff and the closure of infected wards. These outbreaks of highly resistant pathogens reinforce the clinical importance of antimicrobial agents such as tigecycline, daptomycin, linezolid, and vancomycin, which often retain excellent in vitro activity against even highly resistant pathogens [16, 17].

As a result of a resistance control programme started in 2003 in 38 French teaching hospitals, vancomycin-resistant enterococci and carbapenemase-producing Enterobacteriaceae cases were controlled while MRSA incidence declined by two thirds; however, a dramatic increase in the percentage of ESBL-positive Enterobacteriaceae was noted [4]. Similarly, a long-term study involving 933 health care facilities carried out by the French national healthcare-associated infection early-warning, investigation and surveillance network (RAISIN) led to a 43% decrease in MRSA while ESBL-positive Enterobacteriaceae increased by 182% [18]. The epidemiology of ESBL-producing pathogens can be very complex [19], and ESBL-positive Enterobacteriaceae are increasing in prevalence so rapidly that they may soon become the most widespread MDR pathogens in French hospitals [20]. ESBL levels among *E. coli* and *K. pneumoniae* increased markedly over the course of the T.E.S.T. study; however, MRSA levels in the current study decreased between 2004 (34.3%) and 2009 (20.0%) but increased from 2011 (23.4%) to 2012 (34.7%). This increase in MRSA levels was unexpected and may have been due to regional factors such as localised outbreak(s) of resistant isolates.

In a review of data collected by the Pneumococcus Surveillance Network (PSN) in France in 2007, Kempf et al. [21] reported a PRSP percentage of 6.6% among *S. pneumoniae* isolates collected from adults and children. This PRSP occurrence is twice that recorded in the current manuscript for France between 2004 and 2012, and three times higher than the value reported in T.E.S.T. for 2007 alone. This difference is due in part to Kempf et al. [21] using a resistance breakpoint of >1 mg/L for penicillin, compared with ≥4 mg/L used in this T.E.S.T. study. Sizeable (>20%) regional variations in the prevalence of penicillin-non-susceptible *S. pneumoniae* and a high number of isolates collected from children (27.9%) were also reported by Kempf et al. [21].

Tigecycline and linezolid demonstrated good activity against the Gram-positive isolates in this study. In the case of enterococci the activity of tigecycline and linezolid has also been demonstrated by others [15, 22, 23]. Bourdon et al. [15] performed susceptibility testing on 602 *E. faecium* and 30 *E. faecalis* isolates, all VRE, collected from 112 French hospitals between 2006 and 2008 and observed 100% susceptibility to tigecycline and linezolid. Similarly, Marcadé et al. [22] described seven glycopeptide-resistant *E. faecium* isolates from a single hospital in Paris which possessed both *vanA* and *vanB* resistance genes; all were susceptible to tigecycline and linezolid, as well as daptomycin. Bérenger et al. [23] examined 60 glycopeptide-resistant, epidemiologically unrelated clinical isolates of *E. faecium* collected in France between 2006 and 2008; all were susceptible to linezolid while 59 were tigecycline-susceptible (the remaining isolate had intermediate susceptibility for tigecycline).

In the current T.E.S.T. report, the levels of β-lactamase positive isolates of *H. influenzae* fluctuated year-on-year (between 18.1% and 27.7%) between 2004 and 2012, with no discernible pattern over time. A statistically significant decrease in the occurrence of β-lactamase-positive, ampicillin-resistant isolates among non-typeable *H. influenzae* was reported in France between 2001 and 2008 [24], with the rate decreasing from 35.6% in 2001–02 to 13.5% in 2007–08; however, this study only included isolates collected from patients ≤5 years in age.

## Conclusions

Programmes aimed at controlling and/or reducing the prevalence of drug-resistant pathogens in France have been successful against some important pathogens, such as MRSA and VRE, but other resistant pathogens continue to increase in prevalence across the country, most notably ESBL-positive Enterobacteriaceae. These trends highlight the importance of surveillance studies such as T.E.S.T., which monitor pathogen resistance rates against key antimicrobial agents both nationally and globally. Tigecycline possesses good in vitro activity against many resistant pathogens, including ESBL producers, and thus could be a useful tool in the treatment of resistant infections in France in the future.

## Electronic supplementary material

Additional file 1: Table S1: MIC_90_ (mg/L) and antimicrobial susceptibility (%S) of clinically important Gram-positive and Gram-negative isolates. (DOC 312 KB)
