# Supplementary material for: A longitudinal assessment of antimicrobial susceptibility among important pathogens collected as part of the Tigecycline Evaluation and Surveillance Trial (T.E.S.T.) in France between 2004 and 2012
Source: Antimicrob Resist Infect Control. 2014 Dec 1;3:36. doi: 10.1186/2047-2994-3-36 (PMC4322957; doi:10.1186/2047-2994-3-36)
Supplement: Supplementary file 1 — Additional file 1: Table S1: MIC90 (mg/L) and antimicrobial susceptibility (%S) of clinically important Gram-positive and Gram-negative isolates. (DOC 312 KB) [file 13756_2014_601_MOESM1_ESM.doc]

## Additional file 1: Table S1. MIC_90_ (mg/L) and antimicrobial susceptibility (%S) of clinically important Gram-positive and Gram-negative isolates

| Pathogen | 2004 | | 2005 | | 2006 | | 2007 | | 2008 | | 2009 | | 2010 | | 2011 | | 2012 | | Significance |
| --- | --- | --- | --- | --- | --- | --- | --- | --- | --- | --- | --- | --- | --- | --- | --- | --- | --- | --- | --- |
|  | MIC_90_ | %S | MIC_90_ | %S | MIC_90_ | %S | MIC_90_ | %S | MIC_90_ | %S | MIC_90_ | %S | MIC_90_ | %S | MIC_90_ | %S | MIC_90_ | %S | (+/-)^c^ |
| Gram-positive |  | |  | |  | |  | |  | |  | |  | |  | |  | |  |
| *E. faecalis* | n=50 (46/7) | | n=29 (25/4) | | n=65 (42/23) | | n=120 (24/96) | | n=200 (0/200) | | n=180 (0/180) | | n=146 (0/146) | | n=26 (0/26) | | n=153 (0/153) | |  |
| AMC | 0.5 | 100 | 0.5 | 100 | 1 | 100 | 1 | 99.2 | 1 | 99.5 | 1 | 100 | 1 | 98.6 | 1 | 100 | 1 | 96.7 | p<0.01 (+) |
| AMP | 1 | 100 | 1 | 100 | 1 | 100 | 1 | 99.2 | 2 | 99.5 | 2 | 100 | 2 | 97.9 | 1 | 100 | 2 | 95.4 | p<0.001 (+) |
| IMP | 1 | 100 | 4 | 96.0 | 4 | 95.2 | 4 | 91.7 | - | - | - | - | - | - | - | - | - | - | - |
| LZD | 2 | 100 | 2 | 100 | 2 | 100 | 2 | 100 | 2 | 100 | 2 | 100 | 2 | 100 | 2 | 100 | 2 | 100 | - |
| TIG | 0.12 | 100 | 0.12 | 100 | 0.25 | 100 | 0.25 | 100 | 0.25 | 100 | 0.25 | 98.9 | 0.25 | 100 | 0.25 | 100 | 0.12 | 100 | N.S. |
| VAN | 2 | 100 | 2 | 100 | 2 | 98.5 | 2 | 100 | 2 | 100 | 2 | 98.9 | 2 | 99.3 | 2 | 96.2 | 2 | 98.7 | N.S. |
| *E. faecium* | n=9 (9/0) | | n=9 (8/1) | | n=22 (6/16) | | n=41 (6/35) | | n=74 (0/74) | | n=60 (0/60) | | n=55 (0/55) | | n=15 (0/15) | | n=47 (0/47) | |  |
| AMC | - | [7] | - | [3] | ≥16 | 22.7 | ≥16 | 26.8 | ≥16 | 28.4 | ≥16 | 20.0 | ≥16 | 12.7 | ≥16 | 46.7 | ≥16 | 21.3 | N.S. |
| AMP | - | [5] | - | [3] | ≥32 | 13.6 | ≥32 | 22.0 | ≥32 | 25.7 | ≥32 | 20.0 | ≥32 | 12.7 | ≥32 | 40.0 | ≥32 | 21.3 | N.S. |
| LZD | - | [9] | - | [9] | 2 | 100 | 2 | 100 | 2 | 100 | 2 | 100 | 2 | 100 | 2 | 100 | 2 | 100 | - |
| TIG | - | [9] | - | [9] | 0.25 | 100 | 0.25 | 100 | 0.25 | 100 | 0.25 | 100 | 0.25 | 100 | 0.25 | 100 | 0.25 | 100 | - |
| VAN | - | [9] | - | [9] | 1 | 100 | 2 | 95.1 | 8 | 89.2 | 2 | 96.7 | 1 | 94.5 | 2 | 93.3 | 1 | 95.7 | N.S. |
| *S. aureus* | n=99 (88/11) | | n=63 (46/17) | | n=131 (82/49) | | n=297 (51/246) | | n=479 (0/479) | | n=385 (0/385) | | n=287 (0/287) | | n=64 (0/64) | | n=424 (2/422) | |  |
| LEV | 16 | 61.6 | 16 | 69.8 | 8 | 65.6 | 16 | 68.4 | 16 | 72.7 | 16 | 80.0 | 16 | 73.9 | 16 | 75.0 | 16 | 67.9 | N.S. |
| LZD | 4 | 100 | 2 | 100 | 4 | 100 | 2 | 100 | 2 | 100 | 4 | 100 | 4 | 100 | 2 | 100 | 2 | 100 | - |
| MIN | ≤0.25 | 93.9 | ≤0.25 | 93.7 | ≤0.25 | 91.6 | 0.5 | 94.9 | 0.5 | 93.5 | 0.5 | 91.2 | 0.5 | 91.3 | ≤0.25 | 98.4 | ≤0.25 | 96.9 | N.S. |
| PEN | ≥16 | 11.1 | ≥16 | 11.1 | ≥16 | 10.7 | ≥16 | 14.5 | ≥16 | 14.4 | ≥16 | 16.1 | ≥16 | 19.2 | ≥16 | 17.2 | ≥16 | 11.8 | N.S. |
| TIG | 0.12 | 100 | 0.12 | 100 | 0.25 | 100 | 0.25 | 100 | 0.25 | 100 | 0.5 | 100 | 0.5 | 100 | 0.12 | 100 | 0.25 | 100 | - |
| VAN | 1 | 100 | 1 | 100 | 1 | 100 | 1 | 100 | 1 | 100 | 1 | 100 | 1 | 100 | 1 | 100 | 1 | 100 | - |
| *S. agalactiae* | n=43 (42/1) | | n=21 (15/6) | | n=61 (23/38) | | n=111 (8/103) | | n=184 (0/184) | | n=158 (0/158) | | n=129 (0/129) | | n=27 (0/27) | | n=125 (0/125) | |  |
| LEV | 1 | 95.3 | 1 | 100 | 1 | 100 | 1 | 96.4 | 1 | 97.3 | 1 | 98.1 | 1 | 96.1 | 1 | 96.3 | 1 | 96.0 | N.S. |
| LZD | 1 | 100 | 1 | 100 | 1 | 100 | 1 | 100 | 1 | 100 | 1 | 100 | 2 | 100 | 1 | 100 | 1 | 100 | - |
| MIN | ≥16 | 20.9 | ≥16 | 14.3 | ≥16 | 14.8 | ≥16 | 18.9 | ≥16 | 14.7 | ≥16 | 10.1 | ≥16 | 18.6 | ≥16 | 18.5 | ≥16 | 14.4 | N.S. |
| PEN | ≤0.06 | 100 | 0.12 | 100 | 0.12 | 100 | 0.12 | 100 | 0.12 | 100 | 0.12 | 100 | 0.12 | 100 | 0.12 | 100 | 0.12 | 100 | - |
| TIG | 0.25 | 100 | 0.12 | 100 | 0.06 | 100 | 0.12 | 100 | 0.12 | 100 | 0.12 | 100 | 0.25 | 100 | 0.25 | 100 | 0.12 | 100 | - |
| VAN | 0.5 | 100 | 0.5 | 100 | 0.5 | 100 | 0.5 | 100 | 0.5 | 100 | 1 | 100 | 1 | 100 | 0.5 | 100 | 0.5 | 100 | - |
| *S. pneumoniae* | n=52 (48/4; 44^a^) | | n=29 (20/9; 26^a^) | | n=65 (38/27; 60^a^) | | n=153 (14/139; 124^a^) | | n=199 (0/199; 162^a^) | | n=177 (0/177; 171^a^) | | n=148 (0/148; 130^a^) | | n=18 (0/18; 14^a^) | | n=149 (0/149; 141^a^) | |  |
| AMP | 2 | 59.6 | 4 | 72.4 | 2 | 70.8 | 2 | 69.9 | 4 | 61.3 | 2 | 71.2 | 4 | 72.3 | 4 | 55.6 | 2 | 70.5 | N.S. |
| AZI^a^ | ≥128 | 50.0 | ≥128 | 34.6 | ≥128 | 45.0 | ≥128 | 57.3 | ≥128 | 54.9 | ≥128 | 64.3 | 64 | 59.2 | 64 | 50.0 | 64 | 57.4 | N.S. |
| CRO | 1 | 69.2 | 1 | 86.2 | 1 | 84.6 | 1 | 83.0 | 1 | 74.4 | 1 | 78.0 | 1 | 73.6 | 1 | 72.2 | 1 | 88.6 | N.S. |
| CLA^a^ | ≥128 | 50.0 | ≥128 | 34.6 | ≥128 | 45.0 | ≥128 | 57.3 | ≥128 | 56.2 | ≥128 | 65.5 | ≥128 | 59.2 | 64 | 50.0 | 64 | 57.4 | N.S. |
| CLI^a^ | ≥128 | 52.3 | ≥128 | 42.3 | ≥128 | 60.0 | ≥128 | 62.1 | ≥128 | 63.6 | ≥128 | 73.1 | ≥128 | 67.7 | ≥128 | 64.3 | ≥128 | 67.4 | p<0.01 (-) |
| ERY^a^ | ≥128 | 50.0 | ≥128 | 34.6 | ≥128 | 45.0 | ≥128 | 57.3 | ≥128 | 54.9 | ≥128 | 64.9 | ≥128 | 57.7 | 64 | 50.0 | 64 | 57.4 | N.S. |
| IMI | 0.5 | 100 | 0.25 | 100 | ≤0.12 | 100 | 0.25 | 100 | - | - | - | - | - | - | - | - | - | - | - |
| LEV | 1 | 100 | 1 | 100 | 1 | 100 | 1 | 100 | 1 | 99.5 | 1 | 99.4 | 1 | 98.6 | 1 | 100 | 1 | 100 | N.S. |
| LZD | 1 | 100 | 1 | 100 | 1 | 100 | 1 | 99.3 | 1 | 100 | 1 | 100 | 1 | 100 | 1 | 100 | 1 | 100 | N.S. |
| MER | - | [4] | - | [9] | 1 | 100 | 0.5 | 100 | 0.5 | 100 | 0.5 | 100 | 0.5 | 100 | 0.5 | 100 | 0.5 | 99.3 | - |
| MIN | 8 | 55.8 | 4 | 72.4 | 4 | 67.7 | 8 | 62.7 | ≥16 | 35.2 | 8 | 35.6 | ≥16 | 42.6 | ≥16 | 55.6 | 8 | 50.3 | p<0.01 (+) |
| PEN | 2 | 50.0 | 2 | 51.7 | 2 | 60.0 | 2 | 51.0 | 2 | 45.7 | 2 | 55.4 | 2 | 53.4 | 2 | 44.4 | 2 | 49.7 | N.S. |
| VAN | 0.5 | 100 | 0.5 | 100 | 0.5 | 100 | 0.5 | 100 | 0.5 | 100 | 0.5 | 100 | 0.5 | 100 | 0.5 | 100 | 0.5 | 100 | - |
| Gram-negative |  |  |  |  |  |  |  |  |  |  |  |  |  |  |  |  |  |  |  |
| *E. aerogenes* | n=33 (33/0) | | n=12 (12/0) | | n=40 (30/10; 39^b^) | | n=72 (6/66; 70^b^) | | n=113 (0/113) | | n=101 (0/101) | | n=89 (0/89) | | n=23 (0/23) | | n=78 (0/78) | |  |
| AMK | 8 | 90.9 | 8 | 91.7 | 8 | 92.5 | 4 | 91.7 | 8 | 98.2 | 4 | 98.0 | 4 | 96.6 | 8 | 95.7 | 4 | 97.4 | N.S. |
| CFP | 16 | 75.8 | 16 | 75.0 | 2 | 82.5 | 1 | 90.3 | 2 | 86.7 | 1 | 90.1 | 4 | 83.1 | 1 | 91.3 | 1 | 92.3 | N.S. |
| CRO | 16 | 66.7 | 32 | 33.3 | 32 | 35.0 | 16 | 65.3 | 32 | 57.5 | 16 | 65.3 | 64 | 46.1 | 8 | 65.2 | 16 | 56.4 | N.S. |
| IMI | 1 | 100 | 1 | 100 | 1 | 96.7 | - | [5] | - | - | - | - | - | - | - | - | - | - | - |
| LEV | ≥16 | 78.8 | ≥16 | 33.3 | ≥16 | 65.0 | ≥16 | 81.9 | ≥16 | 79.6 | ≥16 | 86.1 | ≥16 | 70.8 | ≥16 | 65.2 | 8 | 75.6 | N.S. |
| MER | - | - | - | - | 0.12 | 100 | 0.25 | 97.0 | 0.12 | 99.1 | 0.12 | 98.0 | 0.25 | 96.6 | 0.12 | 100 | 0.25 | 100 | - |
| PTZ | 16 | 69.7 | 64 | 41.7 | 64 | 45.0 | 64 | 66.7 | 64 | 61.9 | 64 | 61.4 | 128 | 55.1 | 32 | 43.5 | 64 | 62.8 | N.S. |
| TIG | 1 | 97.0 | 2 | 75.0 | 2 | 82.5 | 2 | 84.7 | 1 | 91.2 | 2 | 89.1 | 4 | 82.0 | 2 | 87.0 | 2 | 85.9 | N.S. |
| *E. cloacae* | n=66 (63/3) | | n=45 (45/0) | | n=112 (84/28) | | n=201 (34/167; 198^b^) | | n=375 (0/375) | | n=307 (0/307) | | n=246 (0/246) | | n=48 (0/48) | | n=265 (0/265) | |  |
| AMK | 2 | 98.5 | 4 | 91.1 | 8 | 96.4 | 4 | 98.0 | 4 | 97.3 | 8 | 96.1 | 8 | 95.9 | 8 | 95.8 | 4 | 97.0 | N.S. |
| CFP | 2 | 81.8 | 32 | 46.7 | 16 | 65.2 | 4 | 73.6 | 8 | 68.3 | 8 | 64.8 | 16 | 61.0 | 32 | 62.5 | 32 | 66.4 | N.S. |
| CRO | 64 | 63.6 | ≥128 | 40.0 | ≥128 | 47.3 | ≥128 | 54.2 | ≥128 | 50.9 | ≥128 | 52.4 | ≥128 | 44.3 | 64 | 52.1 | 64 | 51.3 | N.S. |
| IMI | 1 | 100 | 1 | 100 | 1 | 97.6 | 0.25 | 100 | - | - | - | - | - | - | - | - | - | - |  |
| LEV | 4 | 81.8 | ≥16 | 62.2 | ≥16 | 67.0 | 8 | 78.6 | ≥16 | 76.0 | ≥16 | 73.3 | ≥16 | 64.6 | ≥16 | 68.8 | ≥16 | 75.5 | N.S. |
| MER | - | [3] | - | - | 0.12 | 100 | 0.25 | 100 | 0.25 | 98.7 | 0.25 | 100 | 0.25 | 99.2 | 0.25 | 100 | 0.25 | 99.2 | - |
| PTZ | 128 | 68.2 | ≥256 | 42.2 | ≥256 | 55.4 | 128 | 63.7 | ≥256 | 60.5 | ≥256 | 57.0 | ≥256 | 52.8 | 128 | 56.3 | 128 | 66.0 | N.S. |
| TIG | 1 | 92.4 | 2 | 71.1 | 2 | 83.9 | 2 | 86.6 | 1 | 90.1 | 2 | 84.0 | 4 | 74.0 | 2 | 85.4 | 2 | 89.1 | N.S. |
| *E. coli* | n=101 (99/2) | | n=54 (50/4) | | n=173 (126/47) | | n=277 (49/228) | | n=493 (0/493) | | n=429 (0/429) | | n=331 (0/331) | | n=77 (0/77) | | n=349 (0/349) | |  |
| AMK | 8 | 97.0 | 4 | 100 | 4 | 97.7 | 4 | 97.5 | 8 | 97.0 | 8 | 97.2 | 4 | 98.5 | 4 | 100 | 4 | 99.1 | N.S. |
| AMC | 32 | 80.2 | 16 | 83.3 | 32 | 72.8 | 32 | 69.7 | 32 | 69.0 | 32 | 67.1 | 32 | 66.2 | 16 | 81.8 | 16 | 75.4 | N.S. |
| AMP | ≥64 | 55.4 | ≥64 | 55.6 | ≥64 | 38.2 | ≥64 | 40.4 | ≥64 | 38.3 | ≥64 | 36.6 | ≥64 | 35.0 | ≥64 | 45.5 | ≥64 | 33.2 | p<0.001 (+) |
| CFP | ≤0.5 | 97.0 | ≤0.5 | 94.4 | ≤0.5 | 93.1 | 1 | 90.6 | 8 | 80.9 | 16 | 80.2 | 8 | 82.2 | 16 | 83.1 | 8 | 81.7 | p<0.0001 (+) |
| CRO | ≤0.06 | 96.0 | 0.12 | 94.4 | 0.5 | 90.8 | 0.5 | 90.6 | 64 | 81.5 | ≥128 | 79.5 | 64 | 82.8 | 64 | 81.8 | 64 | 81.1 | p<0.0001 (+) |
| IMI | 0.5 | 100 | 0.25 | 100 | 0.5 | 100 | ≤0.06 | 100 | - | - | - | - | - | - | - | - | - | - |  |
| LEV | 0.5 | 92.1 | 4 | 88.9 | ≥16 | 78.6 | ≥16 | 80.1 | ≥16 | 76.3 | ≥16 | 78.8 | ≥16 | 79.8 | 8 | 84.4 | 8 | 81.1 | N.S. |
| MER | - | [2] | - | [4] | ≤0.06 | 100 | ≤0.06 | 100 | ≤0.06 | 99.8 | ≤0.06 | 100 | ≤0.06 | 100 | ≤0.06 | 100 | ≤0.06 | 100 |  |
| PTZ | 2 | 95.0 | 4 | 94.4 | 32 | 87.3 | 16 | 88.4 | 16 | 86.8 | 16 | 87.2 | 16 | 88.8 | 4 | 92.2 | 4 | 92.6 | N.S. |
| TIG | 0.25 | 99.0 | 0.25 | 100 | 0.5 | 100 | 0.5 | 99.6 | 0.5 | 99.4 | 0.5 | 99.1 | 0.5 | 97.9 | 0.25 | 100 | 0.25 | 100 | N.S. |
| *H. influenzae* | n=56 (48/8) | | n=26 (22/4) | | n=62 (49/13) | | n=166 (30/136) | | n=251 (0/251) | | n=224 (0/224) | | n=152 (0/152) | | n=47 (0/47) | | n=207 (7/200) | |  |
| AMC | 1 | 98.2 | 0.5 | 100 | 1 | 98.4 | 1 | 100 | 2 | 99.6 | 1 | 100 | 2 | 97.4 | 2 | 100 | 1 | 99.0 | N.S. |
| AMP^b^ | ≥64 | 76.8 | 16 | 73.1 | 16 | 72.6 | 32 | 80.7 | 32 | 75.7 | 32 | 73.7 | ≥64 | 73.7 | ≥64 | 72.3 | 16 | 76.3 | N.S. |
| CRO | ≤0.06 | 100 | ≤0.06 | 100 | ≤0.06 | 98.4 | ≤0.06 | 99.4 | ≤0.06 | 97.6 | ≤0.06 | 97.8 | ≤0.06 | 100 | ≤0.06 | 100 | ≤0.06 | 97.6 | N.S. |
| IMI | 1 | 95.8 | 0.5 | 100 | 0.5 | 100 | 0.12 | 100 | - | - | - | - | - | - | - | - | - | [7] | - |
| LEV | 0.015 | 100 | 0.015 | 100 | 0.015 | 100 | 0.03 | 100 | 0.03 | 100 | 0.015 | 100 | 0.03 | 100 | 0.03 | 100 | 0.015 | 100 | - |
| MER | - | [8] | - | [4] | ≤0.06 | 100 | 0.12 | 100 | 0.12 | 100 | 0.12 | 100 | 0.12 | 100 | 0.12 | 100 | 0.12 | 100 | - |
| MIN | 1 | 98.2 | 1 | 96.2 | 1 | 90.3 | 2 | 88.0 | 2 | 84.5 | 1 | 91.1 | 1 | 100 | 1 | 97.9 | 2 | 89.4 | N.S. |
| *K. oxytoca* | n=33 (29/4) | | n=18 (18/0) | | n=49 (41/8; 48^b^) | | n=114 (14/100; 111^b^) | | n=137 (0/137) | | n=132 (0/132) | | n=97 (0/97) | | n=22 (0/22) | | n=93 (0/93) | |  |
| AMK | 4 | 100 | 8 | 94.4 | 4 | 100 | 4 | 99.1 | 2 | 99.3 | 4 | 97.7 | 2 | 99.0 | 4 | 100 | 4 | 97.8 | N.S. |
| AMC | 4 | 93.9 | 32 | 72.2 | 16 | 77.6 | 16 | 86.8 | 32 | 73.7 | 16 | 81.8 | 32 | 75.3 | 16 | 86.4 | 16 | 77.4 | N.S. |
| CFP | ≤0.5 | 97.0 | 4 | 83.3 | 2 | 89.8 | 1 | 90.4 | 2 | 86.1 | ≤0.5 | 91.7 | 2 | 88.7 | ≤0.5 | 95.5 | 2 | 86.0 | N.S. |
| CRO | 0.12 | 93.9 | 8 | 77.8 | 4 | 83.7 | 16 | 86.0 | 16 | 79.6 | 2 | 87.9 | 16 | 79.4 | 2 | 86.4 | 8 | 79.6 | N.S. |
| IMI | 0.5 | 100 | 1 | 100 | 0.5 | 100 | ≤0.06 | 100 | - | - | - | - | - | - | - | - | - | - | - |
| LEV | 0.5 | 90.9 | 4 | 83.3 | 0.5 | 95.9 | 1 | 91.2 | 2 | 86.1 | 0.5 | 93.2 | 2 | 88.7 | 0.25 | 90.9 | 1 | 92.5 | N.S. |
| MER | - | [4] | - | - | - | [8] | 0.12 | 98.0 | ≤0.06 | 100 | ≤0.06 | 100 | ≤0.06 | 100 | ≤0.06 | 100 | ≤0.06 | 100 | - |
| PTZ | 2 | 97.0 | ≥256 | 77.8 | ≥256 | 83.7 | ≥256 | 88.6 | ≥256 | 80.3 | 128 | 82.6 | ≥256 | 81.4 | ≥256 | 86.4 | ≥256 | 79.6 | N.S. |
| TIG | 0.25 | 97.0 | 0.5 | 100 | 0.5 | 98.0 | 1 | 93.0 | 1 | 96.4 | 1 | 98.5 | 1 | 90.7 | 0.5 | 100 | 1 | 93.5 | N.S. |
| *K. pneumoniae* | n=66 (65/1) | | n=54 (53/1) | | n=87 (61/26) | | n=163 (31/132; 162^b^) | | n=345 (0/345) | | n=287 (0/287) | | n=233 (0/233) | | n=50 (0/50) | | n=239 (1/238) | |  |
| AMK | 2 | 98.5 | 4 | 100 | 8 | 93.1 | 4 | 98.2 | 4 | 95.7 | 4 | 97.6 | 4 | 95.7 | 4 | 98.0 | 8 | 95.0 | N.S. |
| AMC | 16 | 84.8 | 16 | 77.8 | 16 | 86.2 | 16 | 84.0 | 32 | 69.3 | 32 | 73.5 | 32 | 61.8 | 16 | 72.0 | 32 | 69.5 | p<0.0001 (+) |
| CFP | ≤0.5 | 95.5 | 1 | 90.7 | 1 | 90.8 | 4 | 87.1 | 32 | 76.8 | ≥64 | 81.5 | ≥64 | 73.4 | 32 | 80.0 | ≥64 | 69.9 | p<0.0001 (+) |
| CRO | 0.5 | 90.9 | 4 | 87.0 | 16 | 86.2 | 16 | 82.2 | ≥128 | 76.5 | ≥128 | 81.5 | ≥128 | 67.4 | 64 | 78.0 | 64 | 69.9 | p<0.0001 (+) |
| IMI | 1 | 100 | 0.5 | 100 | 0.5 | 100 | ≤0.06 | 100 | - | - | - | - | - | - | - | - | - | [1] | - |
| LEV | 0.5 | 93.9 | 8 | 83.3 | 1 | 90.8 | 2 | 87.7 | 8 | 83.5 | ≥16 | 84.3 | ≥16 | 72.1 | 8 | 80.0 | 8 | 77.4 | p<0.0001 (+) |
| MER | - | [1] | - | [1] | ≤0.06 | 100 | 0.12 | 100 | ≤0.06 | 99.4 | ≤0.06 | 99.3 | ≤0.06 | 100 | ≤0.06 | 100 | 0.12 | 99.6 | - |
| MIN | 8 | - | ≥32 | - | ≥32 | - | 16 | - | ≥32 | - | ≥32 | - | ≥32 | - | 16 | - | 16 | - | - |
| PTZ | 2 | 95.5 | 8 | 94.4 | 16 | 88.5 | 16 | 87.7 | 64 | 80.6 | 128 | 77.4 | 64 | 71.7 | 64 | 82.0 | 64 | 82.4 | p<0.0001 (+) |
| TIG | 0.5 | 93.9 | 2 | 85.2 | 1 | 92.0 | 2 | 81.0 | 1 | 94.2 | 1 | 92.3 | 4 | 75.5 | 1 | 92.0 | 2 | 84.9 | p<0.01 (+) |
| *S. marcescens* | n=38 (37/1) | | n=22 (21/1) | | n=67 (42/25; 66^b^) | | n=113 (17/96) | | n=183 (0/183) | | n=177 (0/177) | | n=128 (0/128) | | n=31 (0/31) | | n=136 (1/135) | |  |
| AMK | 8 | 94.7 | 2 | 100 | 4 | 97.0 | 4 | 98.2 | 4 | 96.7 | 4 | 96 | 4 | 97.7 | 8 | 93.5 | 4 | 98.5 | N.S. |
| CFP | ≤0.5 | 100 | 1 | 95.5 | 1 | 94.0 | 1 | 95.6 | ≤0.5 | 94.0 | 1 | 93.2 | 1 | 93.0 | 1 | 90.3 | ≤0.5 | 96.3 | N.S. |
| CRO | 1 | 92.1 | 16 | 59.1 | 8 | 83.6 | 8 | 80.5 | 8 | 80.3 | 16 | 77.4 | 8 | 85.2 | 32 | 71.0 | 8 | 80.9 | N.S. |
| IMI | 2 | 97.3 | 0.5 | 100 | 2 | 92.9 | 0.12 | 100 | - | - | - | - | - | - | - | - | - | [1] | - |
| LEV | 0.5 | 100 | 2 | 77.3 | 1 | 94.0 | 2 | 84.1 | 2 | 88.5 | 4 | 78.0 | 2 | 87.5 | 1 | 90.3 | 1 | 93.4 | N.S. |
| MER | - | [1] | - | [1] | 2 | 96.0 | 0.12 | 99.0 | 0.12 | 97.8 | 0.12 | 98.9 | ≤0.06 | 100 | 0.12 | 100 | 0.12 | 98.5 | - |
| MIN | 8 | - | 4 | - | 4 | - | 8 | - | 8 | - | 16 | - | 16 | - | 16 | - | 8 | - | - |
| PTZ | 4 | 97.4 | 32 | 68.2 | 16 | 89.6 | 16 | 88.5 | 8 | 91.3 | 16 | 88.7 | 16 | 89.8 | 32 | 83.9 | 16 | 86.0 | N.S. |
| TIG | 2 | 86.8 | 1 | 95.5 | 1 | 91.0 | 2 | 81.4 | 2 | 83.1 | 2 | 72.3 | 2 | 74.2 | 2 | 61.3 | 2 | 85.3 | N.S. |
| *A. baumannii* | n=53 (53/0) | | n=26 (26/0) | | n=102 (69/33) | | n=142 (19/123) | | n=231 (0/231) | | n=231 (0/231) | | n=193 (0/193) | | n=33 (0/33) | | n=150 (3/147) | |  |
| AMK | 32 | 73.6 | 8 | 92.3 | 32 | 87.3 | 32 | 81.0 | ≥128 | 71.0 | ≥128 | 71.4 | ≥128 | 72.0 | 32 | 69.7 | 64 | 80.0 | N.S. |
| IMI | 1 | 98.1 | 1 | 100 | 2 | 97.1 | 2 | 94.7 | - | - | - | - | - | - | - | - | - | [1] | - |
| LEV | 8 | 64.2 | 8 | 50.0 | 8 | 53.9 | 8 | 52.8 | 8 | 58.4 | ≥16 | 55.8 | ≥16 | 53.9 | 8 | 45.5 | ≥16 | 66.7 | N.S. |
| MER | - | - | - | - | 8 | 84.8 | 16 | 82.9 | 4 | 86.1 | 8 | 83.5 | 8 | 81.9 | 4 | 78.8 | 8 | 83.7 | - |
| ***P. aeruginosa*** | n=80 (79/1) | | n=32 (32/0) | | n=152 (120/32) | | n=214 (28/186) | | n=384 (0/384) | | n=336 (0/336) | | n=263 (0/263) | | n=61 (0/61) | | n=258 (1/257) | |  |
| AMK | 8 | 97.5 | 8 | 96.9 | 16 | 84.9 | 16 | 86.9 | 16 | 86.7 | 32 | 84.8 | ≥16 | 86.3 | 16 | 88.5 | 16 | 88.4 | N.S. |
| CFP | 8 | 90 | 16 | 71.9 | 32 | 75.0 | 32 | 75.2 | 16 | 77.3 | 32 | 78.3 | 32 | 75.7 | 16 | 68.9 | 32 | 80.6 | N.S. |
| CTZ | ≤8 | 93.8 | 16 | 81.3 | ≥64 | 74.3 | ≥64 | 73.4 | 32 | 76.3 | ≥64 | 72.3 | ≥64 | 65.4 | 32 | 82 | 32 | 83.7 | N.S. |
| IMP | 8 | 89.9 | 2 | 93.8 | 8 | 86.7 | 16 | 78.6 | - | - | - | - | - | - | - | - | - | [1] | - |
| LEV | ≥16 | 62.5 | ≥16 | 31.3 | ≥16 | 51.3 | ≥16 | 56.1 | ≥16 | 57 | ≥16 | 61.6 | ≥16 | 58.9 | ≥16 | 57.4 | ≥16 | 63.6 | N.S. |
| MER | - | [1] | - | - | ≥32 | 40.6 | 8 | 75.3 | 8 | 73.7 | 8 | 79.8 | 8 | 76.4 | 8 | 63.9 | 8 | 78.2 | - |
| PTZ | 32 | 87.5 | 64 | 81.3 | ≥256 | 70.4 | ≥256 | 70.1 | 128 | 73.4 | ≥256 | 69.9 | ≥256 | 64.6 | 128 | 72.1 | 128 | 79.8 | N.S. |

AMK, amikacin; AMC, amoxicillin-clavulanate; AMP, ampicillin; CFP, cefepime; CTZ, ceftazidime; CRO, ceftriaxone; IMP, imipenem; LEV, levofloxacin; LZD, linezolid; MER, meropenem; MIN, minocycline; PEN, penicillin; PTZ, piperacillin-tazobactam; TIG, tigecycline; VAN, vancomycin.

MIC_90_ and %S are not given where n<10; instead, numbers of susceptible isolates are given in square brackets.

Results do not exactly match those presented by Rodloff et al [6] due to subsequent addition and deletion of isolates from the T.E.S.T. database.

^a^A sub-population of *S. pneumoniae* isolates were tested for susceptibility to azithromycin, erythromycin, clarithromycin and clindamycin.

Values given in square parentheses refer to the number of isolates tested against imipenem and meropenem, respectively (and, where different, ampicillin [^b^]).

^c^A positive (+) change in significance indicates a decrease in susceptibility, while a negative (-) change indicates an increase in susceptibility; N.S., not significant. A cut-off of p<0.1 was used for statistical significance testing.
